# Transcriptomic pathway analysis of urokinase receptor silenced breast cancer cells: a microarray study

**DOI:** 10.18632/oncotarget.21351

**Published:** 2017-09-28

**Authors:** Pavan B. Narayanaswamy, Tapan K. Baral, Hermann Haller, Inna Dumler, Kshitish Acharya, Yulia Kiyan

**Affiliations:** ^1^ Department of Nephrology, Hannover Medical School, Hannover, Germany; ^2^ Shodhaka Life Sciences Private Limited, Bengaluru, India; ^3^ Institute of Bioinformatics and Applied Biotechnology, Bengaluru, India

**Keywords:** urokinase receptor, breast cancer, pathway analysis, microarray

## Abstract

Urokinase plasminogen activator receptor (PLAUR) has been implicated in a variety of physiological and pathological conditions. The multi-functionality of PLAUR is due to its capacity to interact with many co-receptors to regulate extracellular proteolysis and intracellular signaling. Recent reports are identifying novel functions of PLAUR which were not evident in the past; however, the molecular mechanisms of PLAUR signaling are not completely understood. Here, we have compared the transcriptomes of silencing control (sicon) and PLAUR silenced (PLAURsi) MDA-MB-231 breast cancer cells on treatment with radiation. We isolated RNA from the cells, synthesized cDNA and measured the gene expression changes by microarray. We identified 24 downregulated and 53 upregulated genes, which were significantly (P-value < 0.005) affected by PLAUR silencing. Our analysis revealed 415 canonical pathways and 743 causal disease networks affected on silencing PLAUR. Transcriptomic changes and predicted pathways supported and consolidated some of the earlier understanding in the context of PLAUR signaling; including our recent observations in DNA damage and repair process. In addition, we have identified several novel pathways where PLAUR is implicated.

## INTRODUCTION

High-throughput technologies such as microarray, genome sequencing, mass-spectrometry, and genome-wide association studies have been very helpful in deciphering the differences between normal and cancer cells [1]. Protein profiling has helped us to understand the regulation of a huge number of proteins and also in the prediction of regulatory pathway networks. Despite decades of research on cancer, we are still missing links to its understanding. We try here to merge the high throughput power of microarray analysis and pathway prediction programs for better visualization and understanding of gene interaction networks.

Most of the existing therapies for cancer involve the use of DNA damaging drugs and agents like radiation, which induce DNA lesions interfering with DNA replication and transcription [2]. Even in a normal cell, DNA is constantly at risk of damage due to various endogenous and exogenous factors; it has been predicted that oxidative stress inside a cell can damage the DNA 10,000 times per day [3]. To battle DNA damage, cells have evolved numerous sophisticated repair mechanisms for specific kinds of damage; these mechanisms interact and overlap to maintain genome integrity.

The role of urokinase plasminogen activator receptor (PLAUR) in cancer has been extensively studied and as per our knowledge, there is no data available which documents changes in the transcriptome of PLAUR silenced (PLAURsi) cells. PLAUR is a GPI anchored extracellular receptor and localizes the serine protease activity of its ligand urokinase plasminogen activator (PLAU) on the cell membrane [4]. Binding of PLAU leads to the activation of plasmin and generates a proteolytic cascade involving matrix metalloproteases. Thus, the PLAU/PLAUR system is involved in the regulation and remodeling of the extracellular matrix [5, 6]. PLAUR is a multifunctional receptor and can interact with a variety of co-receptors for intracellular signaling [4].

PLAUR has been shown to regulate the migration and proliferation of pancreatic and breast cancer cells [7, 8]. PLAUR overexpression has been observed in many cancers and is often associated with poor survival and prognosis [9, 10]. Previously it was thought that PLAUR promotes cancer progression by regulation of extracellular proteolysis on the cell surface, but recent studies show that PLAUR is involved in many intracellular mechanisms promoting cell survival. It has been observed that PLAUR overexpressing cells have persistent activation of MAPK kinases, pathways involving tyrosine kinase receptors and also G-protein coupled receptors [4, 5, 10]. In agreement with this we have previously shown that PLAUR regulates the ubiquitin proteasome system during DNA damage response and silencing PLAUR impairs DNA repair [11]. We have also recently demonstrated the role of PLAUR in regulating the homologous recombination (HR) DNA repair pathway in MDA-MB-231 and HeLa cells [12].

In this study, we were interested in observing the transcriptomic changes occurring in irradiated PLAURsi cells. We have used high-throughput experimental data to predict novel molecular pathways and functions, inferred from already existing knowledge from biological databases. The predicted pathways are in accordance with known literature from *in vitro* experiments. Our results can have a substantial impact on the understanding of gene interacting networks.

## RESULTS

**Figure 1:**
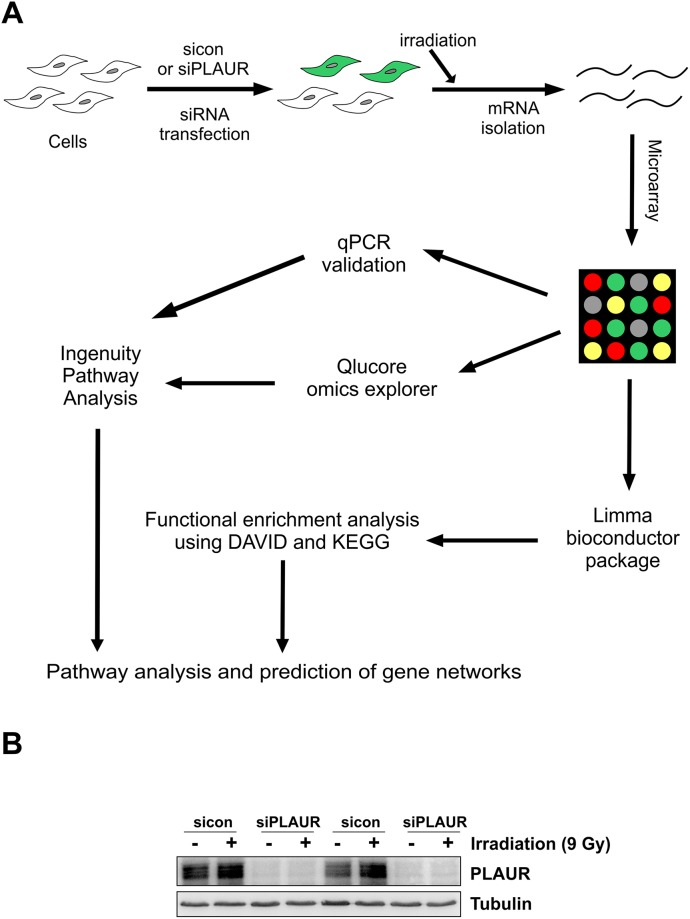
Schematics of microarray analysis on PLAURsi cells **(A)** Flowchart showing the overview of microarray analysis. MDA-MB-231 Cells were transfected with siRNA to silence PLAUR and then irradiated for induction of DNA damage, 4 h later RNA was isolated and hybridized on an Agilent chip in Dual color mode. Raw data obtained after feature extraction was analyzed using Qlucore omics explorer, followed by Ingenuity pathway analysis software (Method 1). Raw data was also used as an input for the limma bioconductor package in R, the shortlisted genes where processed by using DAVID and KEGG databases (Method 2). Both the methods were used to perform pathway analysis and genes were validated by qPCR; **(B)** MDA-MB-231 cells silenced for PLAUR were irradiated at 9 Gy, after 4 h protein lysates were made and subjected to western blotting for the detection of PLAUR.

Microarray data was obtained from sicon and PLAURsi MDA-MB-231 cells, 4 h after irradiation of 9 Gy. This data was processed by two different methods (Figure 1A). The first method involved filtering the genes using Qlucore Omics explorer with a P-value cutoff of 0.05; this resulted in around 370 upregulated and 347 downregulated genes, these genes were further processed by ingenuity pathway analysis (IPA) software to reveal gene interacting networks and functional pathways. The second method involved pre-processing the data using Limma Bioconductor package in R to identify differentially regulated genes using a cutoff P-value < 0.05. Functional enrichment analysis was performed using the Database for Annotation, Visualization and Integrated Discovery (DAVID) for Gene ontology (GO) enrichment analysis and Kyoto Encyclopedia of Genes and Genomes (KEGG) pathway enrichment analysis.

**Figure 2:**
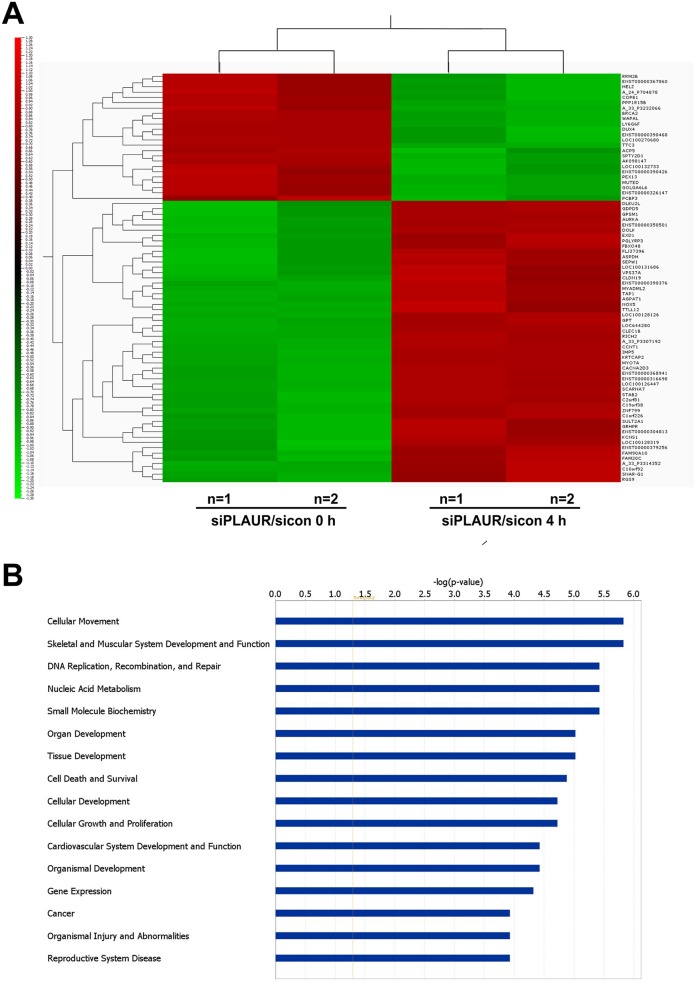
Differentially regulated genes and their functions which are affected on silencing PLAUR **(A)** Heat map depicting the differentially expressed genes with a P-value < 0.005; **(B)** Ingenuity Pathway Analysis of microarray data showing top 16 gene functions which are affected in PLAURsi cells on DNA damage.

Western blotting of lysates from siRNA transfected cells confirmed that there was efficient silencing of PLAUR expression (Figure 1B). We had two parameters to assess, to see the effect of PLAUR silencing alone or effects of PLAUR silencing on induction of DNA damage; the 100 most up- and down-regulated genes in these cases are shown in Supplementary Tables 1-4. We used Qlucore omics explorer to shortlist genes based on a P-value cutoff of 0.005, which resulted in 53 upregulated and 24 downregulated genes; they have been represented as a heatmap (Figure 2A). Table 1 lists the genes in the heatmap along with their biological functions from UniProt [13].

**Table 1:** List of genes represented in the heatmap with known biological functions

	Gene Name	p-value	Fold change	Biological Functions
	TTC3	2.89E-05	-0.84159	protein K48-linked ubiquitination, ubiquitin-dependent protein catabolic process
	LY6G6F	0.00058139	-0.66572	downstream signal transduction pathway
	SPTY2D1	0.00094336	-0.3819215	nucleosome assembly, regulation of chromatin assembly and transcription
Down regulated	BRCA2	0.00105166	-1.21529975	DNA damage response, DNA repair, double-strand break repair via homologous recombination
	WAPAL	0.00108458	-0.066805	cell division, regulation of chromosome segregation and chromatid cohesion
	PEX13	0.00139249	-0.414465	fatty acid alpha-oxidation, cell migration, mitophagy in response to mitochondrial depolarization
	MUTED	0.00140741	-0.1462995	vesicle-mediated transport
	AK098147	0.00144621	-0.284735	unknown function
	PPP1R15B	0.00150601	-0.040375	peptidyl-serine dephosphorylation, endoplasmic reticulum stress-induced eIF2 alpha phosphorylation
	ENST00000390468	0.00231577	-0.0954755	unknown function
	LOC100270680	0.00235518	-1.437085	unknown function
	GOLGA6L6	0.00243931	-0.2633705	unknown function
	ENST00000326147	0.00248927	-0.8985405	unknown function
	A_33_P3232066	0.00266984	-0.0864295	unknown function
	DUX4	0.00284781	-0.43064	negative regulation of G0 to G1 transition, apoptotic process, transcription
	LOC100132733	0.00298165	-0.191127	unknown function
	COPB1	0.0031026	-0.19355765	intracellular protein transport, ER to Golgi vesicle-mediated transport
	ENST00000367860	0.00374651	-0.265195	unknown function
	RRM2B	0.00382213	-0.114055	DNA repair, mitochondrial DNA replication, deoxyribonucleotide biosynthetic process
	HELZ	0.00421811	-0.892889	cellular lipid metabolic process, transcription
	A_24_P704878	0.00422942	-1.1351695	unknown function
	ACP5	0.00453005	-0.46311	bone morphogenesis and resorption, osteoclast differentiation, positive regulation of cell migration
	PCBP3	0.00494525	-0.20857	mRNA metabolic process, nucleic acid binding
	ENST00000390426	0.00498692	-1.15069145	unknown function
	A_33_P3307192	6.56E-05	0.471136	unknown function
	CLEC1B	7.00E-05	0.17857	cell surface receptor signaling pathway, platelet activation and formation
	LOC644280	7.01E-05	0.40716	unknown function
	CCNT1	0.00019679	0.63888	regulation of cell cycle and transcription, regulation of cyclin-dependent protein serine/threonine kinase activity
	LOC100128126	0.00020899	0.932285	unknown function
	KRTCAP2	0.00026415	0.1043485	oligosaccharyltransferase activity
Up regulated	MYO7A	0.00026875	1.213465	actin filament-based movement, intracellular protein transport, lysosome organization
	IMP5	0.00042784	1.555375	protein transport, NLS-bearing protein import into nucleus
	RICH2	0.00043576	0.185575	exocytosis, regulation of small GTPase mediated signal transduction
	LOC100126447	0.00047818	0.840095	unknown function
	SCARNA7	0.00052182	0.57064	unknown function
	GPT	0.00073301	1.126995	cellular amino acid biosynthetic process
	ENST00000316698	0.00073633	0.559268	unknown function
	ZNF799	0.00089184	0.416855	regulation of transcription
	CACNA2D3	0.00094156	0.419785	cardiac conduction, regulation of calcium ion transport
	C19orf38	0.00108418	0.394503	unknown function
	ENST00000368941	0.00108575	1.406455	unknown function
	STAB2	0.00149055	0.913515	angiogenesis, cell adhesion, endocytosis
	TAP1	0.00151008	0.0403275	adaptive immune response, transmembrane transport, antigen processing and presentation
	AURKA	0.00169358	0.196458	cell division, DNA damage response, regulation of cytokinesis
	MYADML2	0.00175261	0.31679	unknown function
	C2orf81	0.00177281	0.1399515	unknown function
	ENST00000350501	0.00182808	0.20949	central nervous system development, positive regulation of GTPase activity, wound healing
	AGPAT1	0.00195538	0.078979	triglyceride biosynthetic process, phospholipid metabolic process
	ENST00000390376	0.00227851	0.255237	unknown function
	C1orf226	0.00241231	0.81491	unknown function
	GPSM1	0.00247166	0.5260285	cell differentiation, regulation of G-protein coupled receptor protein signaling pathway
	C10orf92	0.00256855	0.981	unknown function
	FAM90A10	0.00278555	1.31478	unknown function
	DOLK	0.0027918	0.0563	dolichyl diphosphate biosynthetic process
	A_33_P3314352	0.00286309	0.861685	unknown function
	KCNS1	0.00294728	1.180625	potassium ion transport, protein homooligomerization
	GDPD5	0.00304713	0.280104	lipid metabolic process, positive regulation of cell cycle, cell differentiation
	GRHPR	0.0031282	0.162362	dicarboxylic acid metabolic process, oxidation-reduction process
	SULT2A1	0.00321491	1.008145	3’-phosphoadenosine 5’-phosphosulfate metabolic process, sulfation
	PGLYRP3	0.003229	0.880755	defense response to Gram-positive bacterium, negative regulation of interferon-gamma production
	EXD1	0.0032326	0.206491	gene silencing by RNA, piRNA metabolic process, meiotic cell cycle
	SEPW1	0.00329521	0.1417625	unknown function
	FBXO48	0.00340037	0.4103129	unknown function
	ASPDH	0.00374399	0.570644	NAD biosynthetic process, NADP catabolic process
	FAM20C	0.00381368	0.2310795	biomineral tissue development, osteoclast maturation, positive regulation of bone mineralization
	FLJ37396	0.00381504	1.060099	unknown function
	SNAR-G1	0.00393914	0.64063	unknown function
	RGS9	0.00395577	0.3190388	dopamine receptor signaling pathway, nervous system development, response to estrogen
	DLEU2L	0.00437627	0.168915	unknown function
	VPS37A	0.00441433	0.1255275	autophagy, endosomal transport, protein transport
	ENST00000304813	0.00444994	1.0253	unknown function
	NOX5	0.00445637	0.8472815	angiogenesis, apoptotic process, cell proliferation, cytokine secretion
	TTLL12	0.00445823	0.117902	cellular protein modification process
	LOC100131686	0.00464655	0.2040485	unknown function
	LOC100128319	0.00469658	0.320885	unknown function
	ENST00000379256	0.00485664	0.4476945	unknown function
	CLDN19	0.00491595	0.173423	apical junction assembly, calcium-independent cell-cell adhesion, neuronal action potential propagation

**Figure 3:**
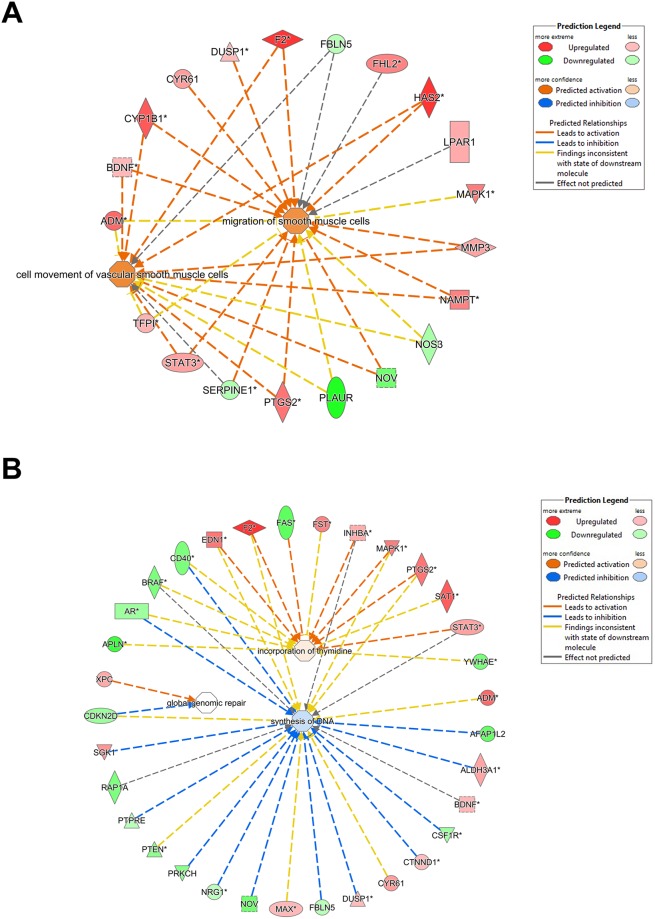
Gene networks affected on silencing PLAUR **(A) and (B)** Gene interacting networks generated using IPA showing the interacting genes and biological processes, cell migration (A) and synthesis of DNA (B).

For pathway prediction analysis we included more number of genes and processed it via IPA software, Figure 2B displays the top 16 gene functions which are affected in PLAURsi cells on DNA damage. The complete list of molecules from our microarray, which can be mapped into these gene functions are shown in Supplementary Table 5. The top gene function affected on silencing PLAUR is predicted to be Cellular Movement and the predicted gene network is shown in Figure 3A. The role of PLAUR in cellular migration is well documented by numerous reports.

**Figure 4:**
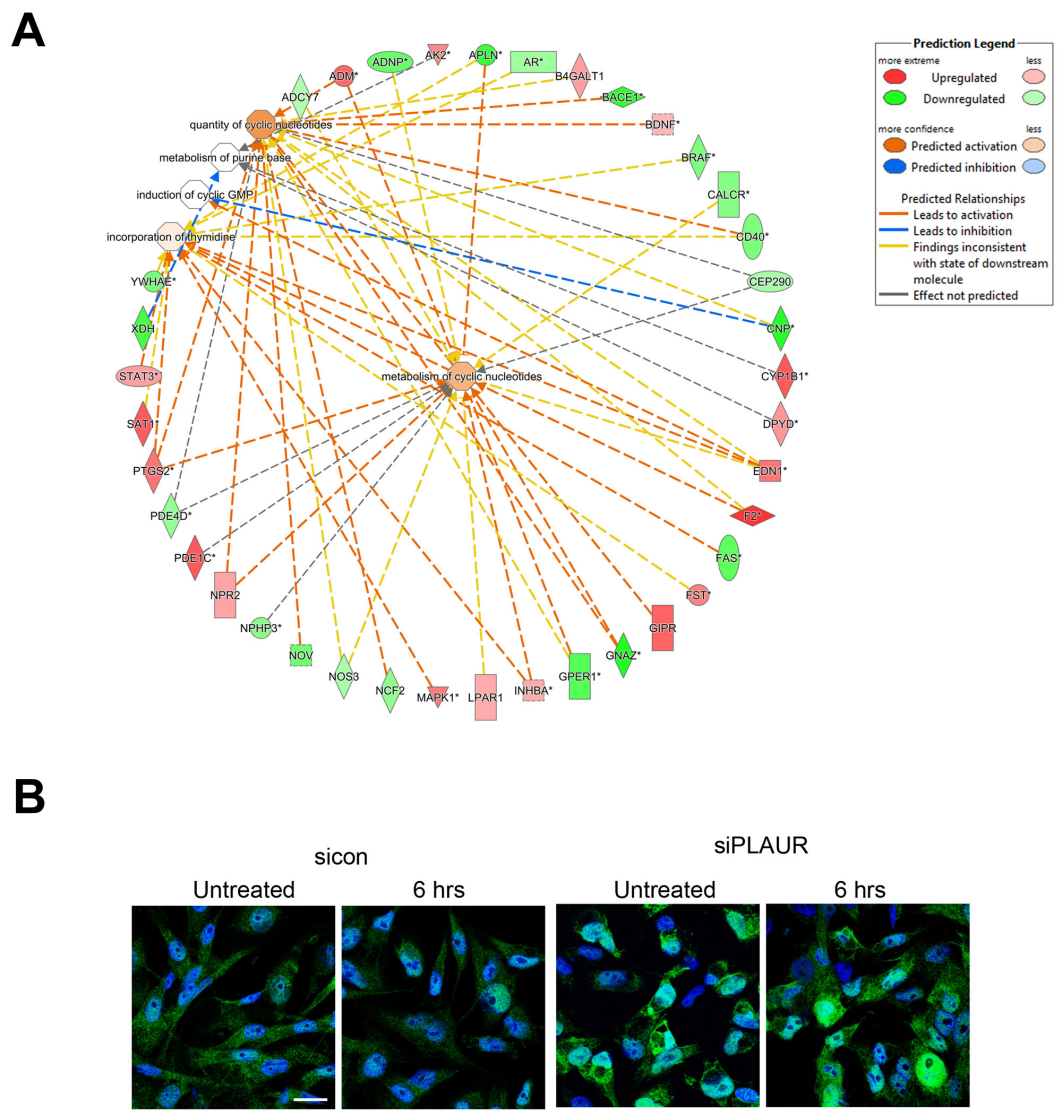
Silencing PLAUR affects proteins involved in DNA metabolism, synthesis and repair **(A)** Gene interacting networks generated using IPA showing the interacting genes and biological processes involving metabolism and incorporation of nucleotides; **(B)** MDA-MB-231 cells silenced for PLAUR were irradiated at 9 Gy, after 6 h cells were fixed and stained for 8-OHdG antibody. DraQ5 was used as nuclear stain. Scale Bar 20 μm.

The gene functions ‘DNA replication, recombination and repair’ and the related ‘Nucleic acid metabolism’ are novel functions of PLAUR which have been investigated by our group. We have demonstrated that PLAUR is essential for DNA damage-induced nuclear import of PSMD6 subunit of 26S proteasome and activation of proteasome activity [11]. Furthermore, we have recently demonstrated that PLAUR is essential for HR repair via activation of CHK-1 and nuclear import of RAD51 protein [12]. Microarray data confirms the involvement of PLAUR in the regulation of DNA metabolism and repair. The gene interacting networks in Figure 3B and 4A displays the complexity involved in regulation of DNA repair by PLAUR. Many of the partners in the network are known to influence the process of DNA synthesis and repair. Regulation of some of these molecules such as STATs, MAPKs and PTEN by PLAUR system is well documented. Whereas, the interrelation of PLAUR with other members of the network awaits further investigation. Differential transcriptional profile of PLAURsi cells suggests impaired DNA repair, and this corresponds to our previous observations [11, 12]. Accordingly, PLAURsi cells demonstrate higher background level of oxidative DNA damage and have more persistent DNA damage after irradiation and 6 h of repair (Figure 4B).

**Figure 5:**
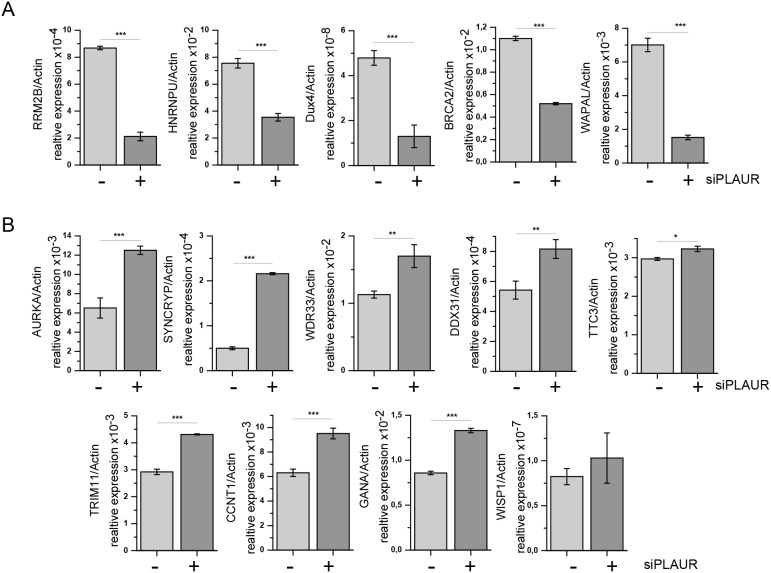
PLAUR regulates mRNA expression of DNA damage related genes MDA-MB-231 cells silenced for PLAUR were treated as for microarray experiment; then RNA was isolated, and SYBR-green RT-PCR for DNA damage-related genes was performed using predesigned KiCqStart^®^ SYBR^®^ Green primers sets (Sigma Aldrich) as described in the Material and Methods. mRNA expression of irradiated sicon and PLAURsi cells are shown as mean±s.d. **(A)** Genes downregulated in irradiated PLAURsi cells in comparison to sicon cells. **(B)** Genes upregulated in irradiated PLAURsi cells in comparison to sicon cells.

**Figure 6:**
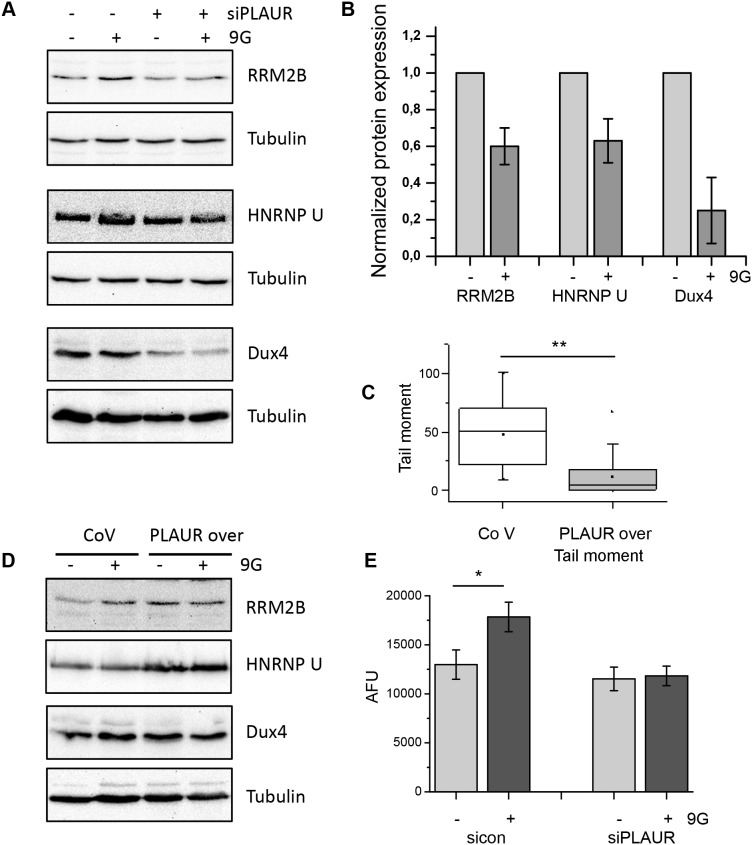
Level of PLAUR expression regulates expression of DNA damage related proteins and efficiency of DNA repair **(A)** MDA-MB-231 cells silenced for PLAUR were treated as for microarray experiment; then western blotting was performed. **(B)** Quantification of the western blotting data from (A) taken from three independent experiments. **(C)** MDA-MB-231 cells infected with control or PLAUR-overexpression lentivirus were treated as for the microarray experiment; then comet assay was performed after 4 h of repair. **(D)** MDA-MB-231 cells infected with control or PLAUR-overexpression lentivirus were treated as above; then western blotting was performed. **(E)** 26S proteasome activity was measured in control and PLAURsi MDA-MB-231 cells 6 h after irradiation.

Further we performed real time PCR to validate some of the important target genes in our microarray. We selected a set of genes among the most regulated genes which were related to the regulation of cell cycle and DNA repair. RT-PCR was performed on irradiated MDA-MB-231 cells using verified KiCqStart primer pairs as described in the Materials and Methods. The RT-PCR data were largely in agreement with the results obtained from our microarray experiment (Figure 5A and 5B). Thus, the expression of RRM2B, HNRNPU, Dux4, BRCA2, and WAPAL in irradiated PLAURsi cells was significantly lower than in irradiated sicon cells. Whereas the expression of SYNCRYP, AURKA, WISP1, WDR33, DDX31, GANA, TRIM11, CCNT1, and TTC3 in irradiated PLAURsi cells was higher than in irradiated sicon cells. Change in expression of some selected genes (RRM2B, HNRNPU, and Dux4) have been also verified by western blotting (Figure 6A). These data were also in agreement with the microarray data.

In our earlier publication, we reported that downregulation of PLAUR expression delays DNA repair in MDA-MB-231 cells and human primary smooth muscle cells [11]. Accordingly, PLAUR overexpression in HEK cells results in increased efficiency of DNA repair [12]. To directly link our data to DNA repair, we overexpressed PLAUR in MDA-MB-231 cells and performed comet assay to assess DNA repair. As shown in Figure 6C, overexpression of PLAUR in MDA-MB-231 indeed improves repair of radiation-induced DNA damage. Further, we tested the expression of some selected proteins from the microarray data. Also, basal level of expression of RRM2B, HNRNPU, and Dux4 were increased in PLAUR-overexpressing cells offering an explanation for improved repair of DNA damage (Figure 6D). However, when analyzing protein expression in PLAURsi cells, one should keep in mind the dependence of proteasome activity on PLAUR expression (Figure 6E). Thus, PLAURsi cells fail to upregulate 26S proteasome activity after DNA damage by irradiation.

**Figure 7:**
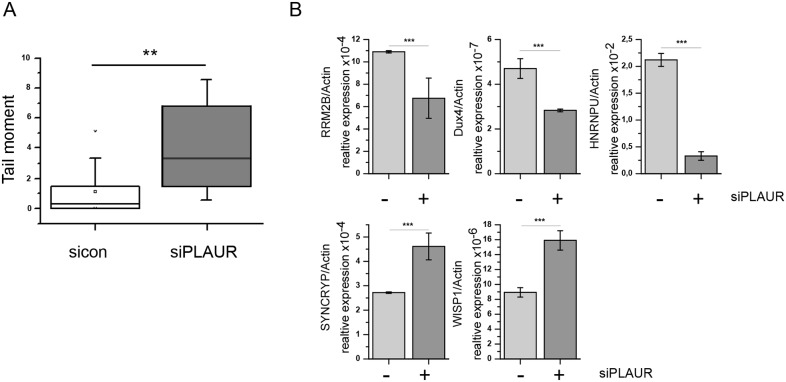
**(A)** HK-2 cells were nucleofected to downregulate PLAUR expression. Then, cells were treated with doxorubicin, and comet assay was performed. **(B)** HK-2 cells silenced for PLAUR were irradiated with 9 Gy. After 4 h RNA was isolated and RT-PCR for DNA damage-related genes was performed using predesigned KiCqStart^®^ SYBR^®^ Green primers sets (Sigma Aldrich) as described in the Material and Methods. mRNA expression of irradiated sicon and PLAURsi cells are shown as mean±s.d.

Further, we wanted to see if these transcriptional effects of PLAURsi can be observed in non-transformed cells. We have shown previously that DNA repair is delayed in PLAURsi human primary smooth muscle cells suggesting that this effect is not cancer cells-specific [11]. Tubular cells are primarily sensitive to nephrotoxic anticancer drugs and we used HK-2 kidney tubular epithelial cells in our experiments. First, we performed comet assay to verify impairment of DNA repair in PLAURsi tubular epithelial cells. As shown in Figure 7A, indeed repair of DNA damage was significantly delayed in PLAURsi HK-2 cells. Next, we analyzed expression of some selected genes identified in the microarray study. We observed that several genes, specifically RRM2B, HNRNPU, Dux4, SYNCRYP, and WISP-1 are regulated in a similar fashion in tubular epithelial cells as in breast cancer cells (Figure 7B) whereas remaining genes have not shown similar regulation (data not shown).

## DISCUSSION

The primary function of PLAUR is to localize the proteolytic activity of PLAU on the extracellular surface and aid in the degradation of extracellular matrix, thereby promoting cell migration [6]. It has been demonstrated that antibodies which block the binding of PLAU to PLAUR affect the migration of endothelial and breast cancer cells [8, 14]. In addition, catalytically inactive PLAU also promotes cell migration via binding to PLAUR and initiation of intracellular signaling mediated by integrins; along with other PLAUR co-receptors in cancer cells, neutrophils, endothelial and smooth muscle cells. PLAUR signaling towards cell movement is mediated by activation of focal adhesion kinase (FAK), myosin light chain kinase (MLCK) and small GTPases of Rho Family [5, 15]. Cell adhesion is also regulated by PLAUR system [16]. PLAUR is highly expressed in majority of cancers and high PLAUR expression correlates with poor patient survival and bad prognosis [17–19]. Genetic knockdown of PLAUR has demonstrated strong anti-tumor activity. However, despite this promising data, therapeutic targeting of PLAUR has not been conceivable. Recently, progress was achieved in targeting PLAUR by monoclonal antibody and small molecule inhibitors (reviewed in [20]). An antibody that showed beneficial anti-cancer effects in preclinical studies was shown to interfere with interaction of PLAUR and integrins [21, 22]. These inhibitory approaches however, work by preventing metastatic dissemination and cannot affect survival of tumor cells.

Recent reports shed light on PLAU/PLAUR roles that are not directly related to cell migration; thus, PLAUR affects epithelial-to-mesenchymal transition [23], stemness properties of cancer cells [24], metabolic state of cancer cells [25]. However, to the best of our knowledge, our work is the first to address transcriptional effects of PLAUR.

Our recent studies describe a new role of PLAUR in facilitating repair of damaged DNA. Our data suggest that high expression of PLAUR is associated with more efficient DNA repair after chemo- or radio-therapy. Specifically, we found homologous DNA DSB repair pathway to be affected by PLAUR [12]. These data offer a new explanation for poor prognosis of cancer patients with high PLAUR expression. To further decipher transcriptional effects of PLAUR in cancer cells, we have performed a microarray study. Pathway analysis highlighted many already well-described roles of PLAUR, such as inflammation [26–30], cardiovascular events [31–33] and liver function [34–36]. Our microarray data has also highlighted a specific function of PLAUR in the kidney that was also documented in the literature (reviewed in [37, 38]).

Further, we selected DNA damage and repair-related genes from the set of most regulated genes and verified their expression by RT-PCR. A set of genes including RRM2B, HNRNPU, BRCA2, and Dux4 were confirmed to be downregulated in PLAURsi cells in comparison to sicon MDA-MB-231 cells. RRM2B, a p53-dependent ribonucleotide reductase subunit B, is a stress response protein upregulated by oxidative stress and DNA damage [39]. Mutations of RRM2B are associated with mitochondrial DNA depletion. Accordingly, mouse embryonic fibroblasts isolated from RRM2B -/- mice showed depletion of mitochondrial DNA [40]. RRM2B protects cell from the oxidative stress by various mechanisms. Thus, it has intrinsic catalase activity [41]. In addition, anti-oxidative function of RRM2B is mediated via direct interaction with Pyrroline-5-carboxylate reductases 1 and 2 (PYCR1, PYCR2) [42]. This complex is essential to maintain mitochondrial integrity and regulates metabolic shift in cancer cells towards cell proliferation [42]. A recent report highlighted its essential role for the reduction of ribonucleotides to deoxyribonucleotides, under hypoxic conditions [43]. Thus, RRM2B provides cancer cells with the ability to replicate DNA and therefore avoid accumulation of DNA damage in hypoxia. RRM2B -/- mice develop severe glomerular damage and kidney insufficiency and die by the age of 8 weeks, due to dNTP pool depletion and p53 activation in the kidney [44, 45]. Hence, decreased RRM2B expression in PLAURsi cells can negatively regulate survival of cancer cells and provide a strong link of PLAUR to kidney damage pathways.

HNRNPU, also called scaffold attachment factor A (SAF-A) is a member of the heterogeneous ribonucleoprotein family involved in a multiplicity of cellular processes such as mRNA turnover, transport, splicing, transcription, protein translation and mitosis [46, 47]. During DNA damage, HNRNPU is rapidly phosphorylated by DNA-PK and this is an essential mechanism for temporal regulation of various DNA repair pathways [48]. Thus, HNRNPU phosphorylation leads to temporal delay of base exchange repair (BER) pathway via displacement of DNA glucosidases thus allowing DNA-PK-mediated NHEJ to take place first [49]. Further, it is recognized that HNRNPU works as a part of anti-DNA:RNA hybrids mechanism [50, 51] to arrest transcription in the proximity of DNA breaks. Since PLAUR silenced cells show low HNRNPU expression after DNA damage, one could expect it to result in accumulation of DNA damage in the absence of PLAUR via different mechanisms.

Double homeobox 4 (DUX4) transcription factor is a main player in development of facioscapulohumeral (FSHD) dystrophy, one of the most common muscular dystrophies. It induces oxidative damage and constitutive DNA damage in FSHD myoblasts [52]. In mammals it is expressed in the testis and epigenetically repressed in most of the tissues [53]. DUX4 activity was recently detected in acute lymphoblastic leukemia [54]. Further, in a recently characterized subset of high-grade sarcoma, so called CDS sarcoma a CIC-DUX4 gene fusion was detected [55]. *in vitro*, CIC-DUX4 exhibited transformed potential in fibroblasts through modification of a cells transcriptome [55]. Furthermore, DUX4 regulates cell cycle arrest through upregulation of p21 expression [56].

BRCA2 or the breast cancer susceptibility gene 2 is a tumor suppressor, which when mutated increases the risk of breast cancer [57]. BRCA2 plays a very important role in the error-free HR repair pathway; it is responsible for loading the recombinase RAD51 onto damaged DNA, where it aids in the search for homologous sequences. We have recently demonstrated that silencing PLAUR affects phosphorylation of RAD51 and impairs HR pathway in cancer cells [12]. Downregulation of BRCA2 after DNA damage can affect RAD51 function and impair the HR pathway [58]. WAPAL is a subunit of the protein complex cohesin, which mediates sister chromatid cohesion during cell division. Cohesin is required for HR mediated DNA double strand break repair (reviewed in [59]). WAPAL is responsible for the dissociation of the cohesin complex [60], and hence may affect DNA repair. It is also known that cohesin recruitment occurs during late G2 phase to repair DNA strand breaks [61]. Therefore, WAPAL downregulation in PLAURsi cells can also contribute to the impaired DNA repair. TTC3 is an E3 ubiquitin ligase which mediates degradation of phosphorylated AKT [62]. The AKT pathway regulates cell cycle progression by regulating purine nucleotide synthesis and affects the G1/S transition [63]. Furthermore, recent reports demonstrated that radiation caused nuclear import of activated AKT and its association with DNA-PK at DSB that potentiated DNA repair [64]. Also, inhibition of AKT pathway is shown to sensitize cancer cells to DNA damaging agents and radiation [65, 66]. CDK9/cyclin T1 complex is a key regulator of RNA polymerase II activity [67] and provides for expression of multiple anti-apoptotic genes. They are mainly recruited to particular promoter sequences and regulate transcription of specific genes [68]. AURKA is known to deregulate DDR and is overexpressed in tumors with genomic instability [69]. DDX31 is an RNA binding protein which is not well studied. Downregulation of DDX31 results in p53 stabilization and apoptosis [70], therefore high expression can lead to genomic instability. TRIM11 (tripartite motif-containing protein 11), is an E3 ubiquitin ligase and high expression of TRIM11 correlates with malignant glioma cells. Knockdown of TRIM11 inhibits proliferation and migration of glioblastoma cells; it's also necessary for the activation of EGFR and MAPK pathways [71].

Hence, the deregulation of a set of DNA damage and repair pathway-associated genes in PLAURsi cells can have a consequence on the downstream DDR signaling, ultimately leading to impairment of DNA repair processes. The top canonical pathways that are affected in PLAURsi cells on irradiation are listed in Table 2. The top toxic functions associated with deficiency of PLAUR on DNA damage are shown in Table 3.

**Table 2:** List of the genes in most significantly affected canonical pathways

Ingenuity Canonical Pathways	-log(p-value)	Ratio	Molecules
Corticotropin Releasing Hormone Signaling	3.91E00	1.23E-01	PRKACB, MAPK1, BDNF, NOS3, MAPK11, RAP1A, BRAF, CALM1 (includes others), PRKCH, MEF2C, PTGS2, NPR2, ADCY7
IL-22 Signaling	2.83E00	2.08E-01	MAPK1, IL10RB, IL22RA1, STAT3, MAPK11
Coagulation System	2.09E00	1.43E-01	PLAUR, F13A1, TFPI, SERPINE1, F2
Leukocyte Extravasation Signaling	1.96E00	7.25E-02	MAPK1, MMP3, ARHGAP4, MAPK11, RAP1A, MMP24, ARHGAP5, WIPF1, NCF2, PRKCH, VCL, ACTG2, MMP1, CTNND1
RhoA Signaling	1.92E00	8.33E-02	ARHGAP5, PLXNA1, MPRIP, CFL2, LPAR1, MYLK2, MYL5, ARHGAP4, PIP5KL1, ACTG2
CMP-N-acetylneuraminate Biosynthesis I (Eukaryotes)	1.91E00	4E-01	NAGK, NANP
cAMP-mediated signaling	1.9E00	6.94E-02	PRKACB, MAPK1, CAMK1D, STAT3, RAP1A, AKAP11, PDE1C, BRAF, CALM1 (includes others), GPER1, LPAR1, DUSP1, PDE4D, PKIA, ADCY7
Cellular Effects of Sildenafil (Viagra)	1.82E00	8.06E-02	PRKACB, CALM1 (includes others), MPRIP, MYL5, MYH3, PDE4D, ACTG2, NOS3, ADCY7, PDE1C
Protein Kinase A Signaling	1.79E00	5.96E-02	PRKACB, PTPRE, MAPK1, YWHAE, PTPN2, MYLK2, MYL5, NOS3, RAP1A, PTEN, AKAP11, PDE1C, BRAF, CALM1 (includes others), PTPRU, PTPRJ, DUSP1, PDE4D, PRKCH, PTGS2, ADCY7, EBI3
UVC-Induced MAPK Signaling	1.76E00	1.19E-01	BRAF, MAPK1, PRKCH, SMPD3, MAPK11
Extrinsic Prothrombin Activation Pathway	1.73E00	1.88E-01	F13A1, TFPI, F2
Fatty Acid α-oxidation	1.73E00	1.88E-01	PTGS2, ALDH3A1, ALDH7A1

**Table 3:** Top toxic functions and diseases implicated on DNA damage in PLAUR silenced cells

Category	p-value	Molecules
Nephrosis	6.13E-04-3.63E-02	CEP290, CEP83, PAX8, NPHP1, NPHP3
Kidney Failure	2.12E-03-5.4E-01	ADM, DACT3, XDH, AGA, TRPV1, NOS3, F2, MR1, AR, EDN1, DUSP1, CD274, PDE4D, PTGS2, CYR61, TFPI, SERPINE1, NPR2, TNS1
Renal Inflammation	2.81E-03-3.97E-01	TRAF3, XDH, DGAT2, TRPV1, NOS3, FAS, FAN1, IFIH1, HRH1, PTGS2, TLR3, SERPINE1, TNS1
Renal Nephritis	2.81E-03-3.97E-01	TRAF3, XDH, DGAT2, TRPV1, NOS3, FAS, FAN1, IFIH1, HRH1, PTGS2, TLR3, SERPINE1, TNS1
Glutathione Depletion In Liver	1.29E-02-3.59E-01	GSTT2/GSTT2B, XDH, CYP1B1, PTEN
Renal Damage	1.57E-02-6.18E-01	ADM, EDN1, DUSP1, PLAUR, TLR3, NOS3, SERPINE1, FAS
Renal Tubule Injury	1.57E-02-1.57E-02	EDN1, PLAUR, SERPINE1
Liver Damage	1.79E-02-5.57E-01	ADM, STAT3, NOS3, INHBA, FAS, F2, PTEN, EDN1, CD40, DUSP1, HLA-DRA, IFNLR1, CD274, PTGS2, TLR3, SERPINE1, ALDH3A1, EBI3
Liver Inflammation/Hepatitis	1.79E-02-6.04E-01	TRAF3, PLAUR, STAT3, FAS, PTEN, PTPRJ, IL10RB, IFNLR1, CD274, PDE4D, TLR3, CYR61, TFPI, SERPINE1, CASP7
Cardiac Inflammation	1.92E-02-1.92E-02	TRAF3, TLR3, NOS3, SERPINE1, CSF1R
Liver Hemorrhaging	1.99E-02-3.63E-02	ADM, DGCR8, PTGS2, FAS
Cardiac Hypertrophy	2.02E-02-6.04E-01	ADM, MAPK1, MYLK2, STAT3, NOS3, INHBA, PTEN, BRAF, ANKRD1, FHL2, EDN1, DUSP1, APLN, MEF2C, PTGS2, ACTG2, SERPINE1, NPR2, MMP1
Glomerular Injury	2.02E-02-5.4E-01	ADM, MAPK1, DACT3, XDH, AGA, TRPV1, TRPC6, NOS3, RAP1A, EDN1, B4GALT1, DUSP1, PTGS2, SERPINE1, TNS1
Renal Proliferation	2.17E-02-4.03E-01	FAT4, SAT1, PLAUR, STAT3, NOS3, ZBTB5, CSF1R, EDN1, DUSP1, APLN, CALCR, HAS2, PTGS2
Cardiac Necrosis/Cell Death	2.23E-02-2.71E-01	ADM, MAPK1, XDH, STAT3, NOS3, MAPK11, INHBA, FAS, PTEN, NRG1, EDN1, APLN, NAMPT, CYR61

Gene ontology analysis using DAVID depicts the enrichment of genes in cellular functions as shown in Supplementary Table 6. The functions include cellular organization, response to cytokines, tissue remodeling, heart development, WNT signaling, adhesion and more; the results also show that nucleobase, nucleoside and nucleotide catabolic processes are affected. These functions directly relate to our results from IPA (Figure 3B and 4A) which shows that PLAURsi cells have defects in DNA synthesis, which can impact the cells ability to replicate DNA and resolve DNA damage.

It is an interesting issue, how a plasma membrane receptor like PLAUR can affect DNA repair events. Our recent mechanistic studies suggested that PLAUR is essential for bystander effect signaling and senses damage-associated molecule(s) released by the irradiated cells [12]. This assumption can be supported by the reports that known PLAUR-signaling network proteins like STAT3 and MAPK promote DNA repair [72–74]. The microarray data presented here confirms our previous observations and identifies several additional potential molecular mechanisms of PLAUR-related DNA metabolism and repair. In addition, many of the predicted biological processes and molecular functions are confirmed by mechanistic studies from our group and others. Furthermore, data from IPA were in agreement with results from DAVID and KEGG, strengthening our data. Our study aids in finding new roles for a cell surface receptor and in developing new diagnostic and therapeutic approaches.

As shown in Table 3, PLAUR is strongly connected to various mechanisms of kidney pathology as well as in inflammation, toxicity of liver and cardiac tissues. Some of those mechanisms are already known whereas others await further investigations [26, 37]. Based on our data, one can assume PLAUR is involved in resolving DNA damage caused by endogenous cues, inflammation [75], chemotherapy or radiation bystander effects thus affecting cells fate and survival.

Our microarray data and pathway analysis confirmed the connection of PLAUR to several reported cell mechanisms, including our recent observations linking PLAUR to DNA repair. This data awaits further investigation aiming at the deciphering the underlying mechanisms and the development of new therapeutics.

## MATERIALS AND METHODS

### Cell culture

Cell line MDA-MB-231 was purchased from ATCC. Cells were tested and authenticated by morphology and western blotting for specific markers in our laboratory. MDA-MB-231 was cultured in DMEM (Lonza) supplemented with 10% FBS and pen strep. Cell lines were tested for mycoplasma contamination every six months.

DNA damage was induced by irradiating the cells using a GammaCell 2000, having Caesium-137 as the radioactive source. The radiation dose was decided based on our previous publication, where we studied the regulation of the DNA damage response and repair pathways by the urokinase receptor [12]. The kinetics of the formation and resolution of p-H2AX foci (which indicates sites of DNA damage) gave us a clear idea of the extent of DNA damage induced by our treatment. We tested different doses of radiation and performed kinetics of some of the important DNA repair proteins, and selected the dose of 9 Gy - which sufficiently elicited the DNA damage response and expression of important DNA repair proteins around the timepoint of 4 h. The effects arising due to radiation induced damage have been extensively studied. Ionising radiation results in numerous kinds of DNA lesions which are chemically caused to the generation of free oxygen species. It has been shown by Cadet et al [76] that ionising radiation creates clustered damage sites on the DNA, corresponding to around 850 pyrimidine lesions, 450 purine lesions, 1000 single-strand breaks (SSB) and 20-40 double-strand breaks (DSB)/cell/Gy.

HK-2 tubular epithelial cell line was purchased from ATCC and cultured as recommended by the supplier.

### Transfection and viral infection

Scrambled control or PLAUR siRNA were obtained from Santa Cruz and transfected using Amaxa Nucleofector™ (Lonza) as previously described. Media was changed after 24 h and cells were used for the experiments 48 h after transfection.

Lentiviruses having a VSV-G envelope were produced using HEK-293T cells. Viral titer was determined by LV Lentiviral Titer kit (MoBiTec) and viruses were used at a multiplicity of infection (MOI) of 1 - 5 using polybrene at a concentration of 2 μg/ml. PLAURsi and Flag-PLAUR overexpression viruses were described earlier [11, 12].

### Western blotting

Western blotting was performed as previously described [12]. Briefly, cells were lysed in RIPA buffer containing protease inhibitors and subjected to sonication. Lysates were then centrifuged at 10,000 rpm for 10 min, the supernatant was collected and protein was estimated by Bradford Reagent (BioRAD). Around 50 μg of protein was boiled with SDS loading buffer and run on a polyacrylamide gel. Proteins were transferred to a PVDF membrane, blocked with 3% BSA and incubated with Primary antibodies. Secondary antibodies conjugated to HRP were used to detect the proteins. The following antibody were used: PLAUR (#3937) from American Diagnostics. Tubulin (#2128) from Cell Signaling Technology, RRM2B (ab8105) from Abcam, HNRNP U (3G6) from Santa Cruz Biotechnology; Dux4 (NBP-49552SS) and TTC3 (NBP1-84293) from Novus Biologicals; WISP1 (AF1627) from R&D Systems; OHdG (bs-1278R) was from Bioss.

### Comet assay

Comet assay was performed as described [77] with some modifications. Briefly, cells were stimulated with γ-radiation and allowed to repair DNA for 4 hrs. Cells were further trypsinised and counted. Approximately 10,000 cells were mixed with 1% low melting agarose and spread on normal agarose pre-coated glass slides. Agarose was allowed to solidify for 30 min at 4 °C, slides were incubated in lysis buffer (1% Triton X-100 in 10mM Tris, 100 mM EDTA) for 4 h, at 4 °C. The slides were incubated in alkaline running buffer (10N NaOH, 200 mM EDTA, pH>13) for 20 min, before performing electrophoresis for 20 min at 300 mA, 25 V. After electrophoresis the slides were 2×5 min incubated with neutralization buffer (0.4M Tris, pH-7.5), placed in cold 100% ethanol for 5 min and dried overnight at 4 °C. Staining was performed with vista green dye (Cell Biolabs) and comets were observed under a fluorescence microscope. Analysis of comet tails was performed by CASP (CaspLab) Software. 50-100 cells were used for quantification of comet tails.

### Immunofluorescence

Immunofluorescence was performed on cells grown overnight on coverslips and then treated with γ-radiation; they were then fixed with 2% formaldehyde at the required time points, permeabilized with 0.1% Triton X-100 and blocked with 3% BSA in PBS at 4 °C overnight. Cells were labeled with primary and corresponding Alexa Fluor^®^ 488- or Alexa Fluor^®^ 594-conjugated secondary antibody (Invitrogen) for 1 h at room temperature. DRAQ5™ (Biostatus) was used for nuclear staining. Cells were then mounted with Aqua-Poly-Mount mounting medium (Polysciences) and analyzed on a Leica TCS-SP2 AOBS confocal microscope. Antibody for 8-OHdG (bs 1278R) was from Bioss.

### Proteasome activity assay

Total proteasome activity in cell lysates was measured using the 20S proteasome assay kit (Cayman Chemical Company, Ann Arbor, MI, USA) as described by the manufacturer. In brief, cells were treated with γ-radiation. The cell lysates were incubated with 10 μM substrate (SUC-LLVY-AMC) for 1 h at 37 °C, the fluorescence was read using a Magellan GENIOUS (Tecan, Männedorf, Switzerland) at 360 nm (excitation) and 480 nm (emission). The enzymatic activity was normalized to the protein concentration. The results are reported as mean ± SD.

### Microarray and data analysis

sicon and PLAURsi MDA-MB-231 cells were treated with radiation of 9 Gy. RNA was prepared after 4 h, by Qiagen RNeasy kit; a technical replicate was included. Microarray data was then generated by the Research Core Unit Transcriptomics (RCUT) of the Hannover Medical School. Briefly, RNA quality was assessed by RNA 6000 Nano Kit assay (Agilent Bioanalyzer 2100). RNA samples from sicon and PLAURsi cells were hybridized on an Agilent chip (Whole Human Genome Oligo Microarray v2, 4×44K) in Dual color mode according to recommended protocols in the ‘Dual-Color Microarray-Based Gene Expression Analysis Protocol V5.7’. Slides were scanned on the Agilent Micro Array Scanner G2565CA (pixel resolution 5 μm, bit depth 20). Data extraction was performed with the ‘Feature Extraction Software V10.7.3.1’. Extracted data was further processed using Omics Explorer software v3.0 (Qlucore) and the genes were filtered using P-value to generate a heat map. The filtered genes were used to perform pathway analysis using IPA (Ingenuity Pathway Analysis) software; functional and causal networks were generated using the information available in the Ingenuity Knowledge Base.

Since the methods used for data analysis are likely to influence the final observation, primary data files obtained after ‘Feature Extraction’ were further analyzed independently by another method. The datasets were subjected to noise reduction, normalization, and differential gene expression analysis. Limma Bioconductor package in R was used to perform data pre-processing and differential gene expression analysis of the microarray dataset (Agilent Dye-swap) [78]. The background noise was eliminated (using normexp method with an offset value=16) and the spot intensity values of both PLAURsi and sicon were converted to a log2 scale. The arrays were then normalized using Loess normalization method; this was followed by the Empirical Bayes method to identify differentially expressed genes between PLAURsi and sicon. The differentially expressed genes identified were screened using a cutoff P-value < 0.05. The probe IDs were converted into gene names based on the platform Whole Human Genome Oligo Microarray v2 (4×44K).

Functional enrichment analysis was performed by submitting the differentially expressed genes as input to DAVID for GO enrichment analysis and KEGG pathway enrichment analysis [79]. The data was screened with the cutoff P-Value < 0.05 in order to identify genes involved in functional pathways.

Microarray data are available in the ArrayExpress database (www.ebi.ac.uk/arrayexpress) under accession number E-MTAB-5298.

### Quantitative RT-PCR analysis

Total RNA was isolated from MDA-MB-231 cells, 4 h after irradiation of 9Gy using RNeasy miniprep kit (Qiagen). Real-time quantitative RT-PCR was performed on a LightCycler^®^ 480 Real-Time PCR System. SYBR Green RT-PCR was performed using Applied Biosystems master mix. Predesigned KiCqStart^®^ SYBR^®^ Green primers were purchased from Sigma. Oligonucleotide sequence is given in the Supplementary Table 7.

## SUPPLEMENTARY MATERIALS FIGURES AND TABLES















## Abbreviations

(PLAUR): Urokinase plasminogen activator receptor
(PLAU): Urokinase plasminogen activator
(GPI): Glycosylphosphatidylinositol
(MAPK): Mitogen-activated protein kinase
(IPA): Ingenuity pathway analysis
(DAVID): Database for Annotation, Visualization and Integrated Discovery
(GO): Gene ontology
(KEGG): Kyoto Encyclopedia of Genes and Genomes
(HR): Homologous recombination
(NHEJ): Non homologous end joining
