# Supplementary material for: Transcriptomic pathway analysis of urokinase receptor silenced breast cancer cells: a microarray study
Source: Oncotarget. 2017 Sep 28;8(60):101572–90. doi: 10.18632/oncotarget.21351 (PMC5731897; doi:10.18632/oncotarget.21351)
Supplement: Supplementary file 1 [file oncotarget-08-101572-s001.pdf]

Transcriptomic pathway analysis of urokinase receptor silenced breast cancer cells: a microarray study

SUPPLEMENTARY MATERIALS

Supplementary Table 1: List of the 100 most up-regulated genes on silencing PLAUR.

See Supplementary File 1

Supplementary Table 2: List of the 100 most down-regulated genes on silencing PLAUR.

See Supplementary File 2

Supplementary Table 3: List of the 100 most up-regulated genes on DNA damage and silencing PLAUR.

See Supplementary File 3

Supplementary Table 4: List of the 100 most down-regulated genes on DNA damage and silencing PLAUR.

See Supplementary File 4

Supplementary Table 5: Top gene functions modified on silencing PLAUR.

See Supplementary File 5

Supplementary Table 6: Gene ontology analysis using DAVID on differentially regulated genes from irradiated PLAURsi cells.

See Supplementary File 6

Supplementary Table 7: List of predesigned KiCqStart® SYBR® Green primers (Sigma Aldrich) used for RTPCR

Name	Forward	Reverse
CCNT1	GGCTTATTTGCAACAAGTTC	CAAATGCAGGCTGTTGG
AURKA	CCTACAAAAGAATATCACGGG	CAAGTACTTCTCTGAGCATTG
DDX31	GACAGAATCTTGGATTTGGG	GATAGCAAGACATTCTGTCG
TRIM11	GAGCTATTACAATTCCTCGG	GGGAAAGATGAATAGCAGTG
WDR33	ATTTGTTGTTACGTGGACTC	CCAATATTTCACATATCCTCCG
SYNCRYP	TAGCAAAGTAACAGAGGGTC	TTCAAGAAAGCAAAAGCCTC
GANAB	CTAGGCAAGGATCAAAAGAC	CAGAGTGAGTTTTGAATGTCTC
WISP1	TCATTAAGGAGGGAAGAAG	GTCTTAGACTTGTAGGGGATG
TTC3	ATGGATTGTATAGAGGAAGGAG	CTAAACTGTCCAGTACGAAG
WAPAL	CGAAGATGATGAAGATGATGAC	TTCTGCATTGTTAGTACCAG
DUX4	GAGAACTGCCATTCTTTCC	AATTTCACGGAAGAACAAGG
RRM2B	AGGAGTTTTTAACAGAAGCC	TGTCAGCTACAAACTCAATG
HNRNPU	AAGTTGTGATGATGATTGGC	TATTTCCCTGGATTTTCTGC
ACTB	GACGACATGGAGAAAATCTG	ATGATCTGGGTCATCTTCTC
